# Effects of ablation depth and repair time on the corneal elastic modulus after laser in situ keratomileusis

**DOI:** 10.1186/s12938-017-0311-5

**Published:** 2017-01-17

**Authors:** Xiaojun Wang, Xiaona Li, Weiyi Chen, Rui He, Zhipeng Gao, Pengfei Feng

**Affiliations:** 1 Collage of Mechanics, Taiyuan University of Technology, Yingze west street 79, Taiyuan, 030024 People’s Republic of China; 2 Department of Excimer Laser, Shanxi Eye Hospital, Fudong street 100, Taiyuan, 030002 People’s Republic of China

**Keywords:** Cornea, LASIK, Ablation depth, Elastic modulus, Repair

## Abstract

**Background:**

The biomechanical properties of the cornea should be taken into account in the refractive procedure in order to perform refractive surgery more accurately. The effects of the ablation depth and repair time on the elastic modulus of the rabbit cornea after laser in situ keratomileusis (LASIK) are still unclear.

**Methods:**

In this study, LASIK was performed on New Zealand rabbits with different ablation depth (only typical LASIK flaps were created; residual stroma bed was 50 or 30% of the whole cornea thickness respectively). The animals without any treatment were served as normal controls. The corneal thickness was measured by ultrasonic pachymetry before animals were humanly killed after 7 or 28 days post-operatively. The corneal elastic modulus was measured by uniaxial tensile testing. A mathematical procedure considering the actual geometrics of the cornea was created to analyze the corneal elastic modulus.

**Results:**

There were no obvious differences among all groups in the elastic modulus on after 7 days post-operatively. However, after 28th days post-operatively, there was a significant increase in the elastic modulus with 50 and 30% residual stroma bed; only the elastic modulus of the cornea with 30% residual stroma bed was significantly higher than that of 7 days.

**Conclusions:**

Changes in elastic modulus after LASIK suggest that this biomechanical effect may correlate with the ablation depth and repair time.

## Background

Laser in situ keratomileusis (LASIK) is now a widely performed refractive procedure designed to improve visual acuity by reshaping the surface of the cornea. It involves the use of a microkeratome to create a thin corneal flap followed by excimer laser ablation of the corneal stroma and repositioning of the flap. The central corneal thickness and curvature change in this surgical procedure, and the cornea undergo tissue remodeling after injury, resulting in the alteration of the corneal biomechanical properties [1]. To perform refractive surgery more accurately, it has been proposed that the biomechanical properties of the cornea should be taken into account [2]. Moreover, post-LASIK keratectasia, a rare but severe complication occurs when a surgical procedure is performed with increasing frequency [3]. Although the mechanism of post-LASIK keratectasia is still unclear, it is widely accepted that corneal biomechanical integrity is compromised after microkeratome incisions [4].

As a non-contact tonometer, Ocular response analyser (ORA) was extensively used to assess the cornea’s viscous and elastic properties by and corneal resistance factor respectively in vivo in recent years. Compared to pre-LASIK, corneal hysteresis and the corneal resistance factor decreased significantly post-operatively [5–10]. The largest changes in corneal biomechanical parameters occurred within 1 week after surgery, and these then became nearly stable during the 6-month follow-up period [8]. Microkeratome flap creation combined with deeper stromal ablation had the greatest effect on the ORA applanation signal, indicating corneas that are more readily deformable [11, 12].

Optical interferometry has also been reported to determine the corneal strain after LASIK in vitro. Their results have shown that the thickness of the remaining tissue is very important for the biomechanical behavior of the cornea after areal ablation [13], and strain increases in corneas with microkeratome incisions [14]. In addition, varying LASIK flap depth and side cut angulations affect the strain behavior of the cornea [15].

It’s worth mentioning that, whether ORA or interferometry can’t represent the material properties of the cornea itself, and the relationship has not been established between the measurement parameters from these techniques and classical biomechanical ones. Because of the relative simplicity and low cost, strip testing is the most common experimental technique used to determine the properties of biomaterials. It has also been attempted by several researchers to determine the stress–strain behaviour of the cornea [16–19]. Fang et al. [20] also measured the corneal elastic modulus of the porcine eyes after LASIK using strip testing. However, because of a spherical surface, variation in thickness, and flattening of the originally curved specimen, it makes strip testing to be a less reliable procedure to determine the corneal material properties [21].

The goals of this study was to advance our understanding on how the ablation depth and repair time influence the corneal material properties after LASIK using animal model, and to create an mathematical analysis procedure that may improve the accuracy of corneal strip testing analysis after LASIK.

## Methods

The animals used in this study were treated in accordance with the guidelines proposed by Animal Care and Use Committee, the Shanxi Science and Technology Department.

### Study design

Forty adult New Zealand white rabbits, weighing 3.0–3.5 kg, were included in this study. All animals were healthy and free of ocular disease. Animals were randomly divided into a control group and three investigative groups (ten rabbits in each group). Animals without any treatment were served as normal controls. In the first group, only typical LASIK flaps were created; in the second and third group, the flaps were created and residual stroma bed was 50 or 30% of the whole cornea thickness respectively. Specimens were collected after 7 or 28 days post-operatively. The corneal strips were prepared for biomechanical testing.

### Laser in situ keratomileusis (LASIK)

Prior to the LASIK, animals underwent the following ocular examinations. Intraocular pressure and central corneal thickness were measured using an ultrasound pachymeter. Ocular anterior and posterior segment lesions would be excluded by slit-lamp biomicroscopy and ophthalmoscope examination. The surgery was performed under general anesthesia produced by intravenous injection from ear edge with 2% pentobarbital sodium. Surface anaesthesia with 0.4% oxybuprocaine was applied to each eye before LASIK.

**Fig. 1:**
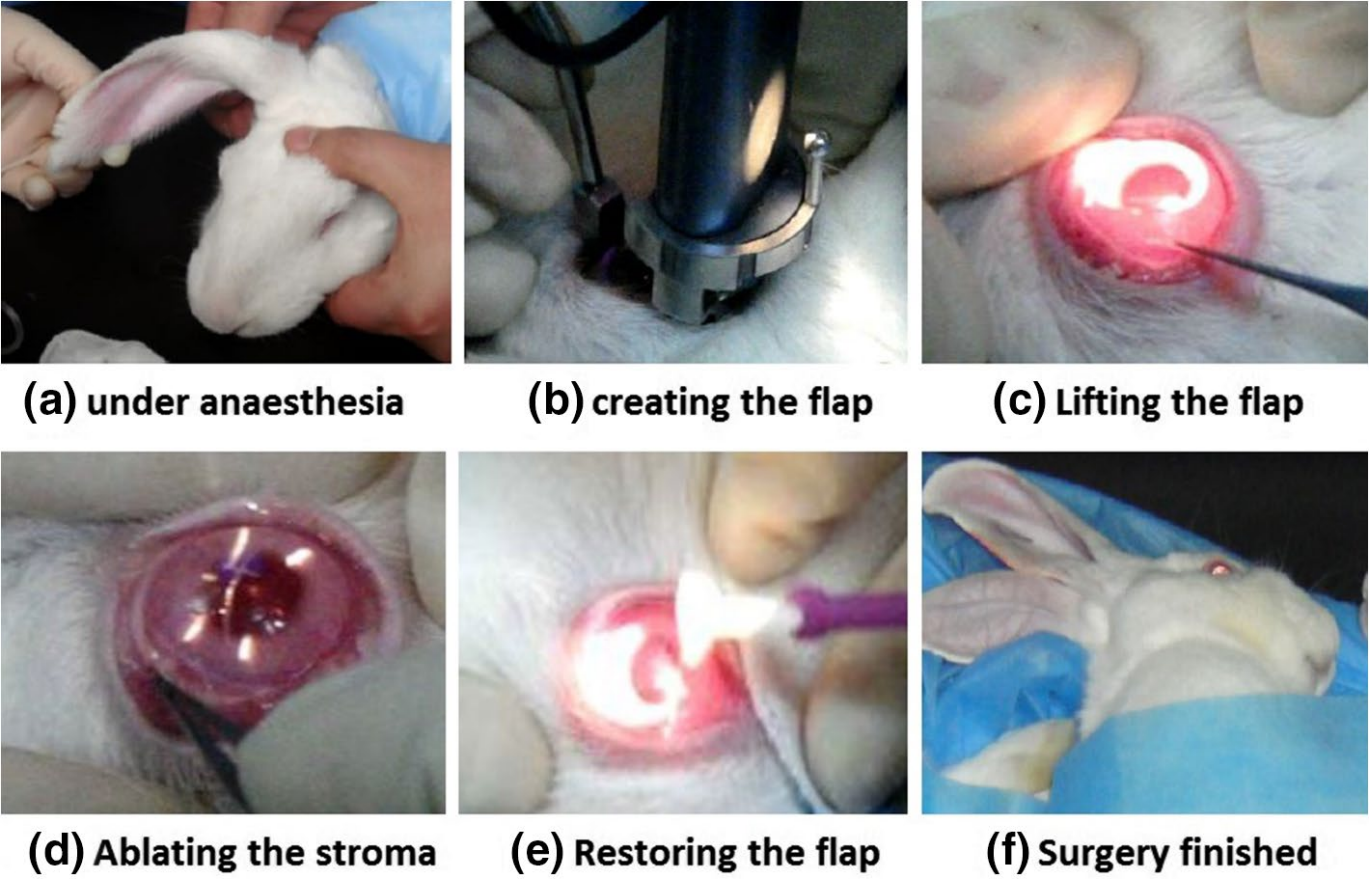
LASIK surgery was performed on a rabbit **a** under anaesthesia, **b** creating the flap, **c** lifting the flap, **d** ablating the stroma, **e** restoring the flap, **f** surgery finished

With the animal under general and local anesthesia, flap (7 mm in diameter and 90–110 μm in thickness) was created by the microkeratome (KM-5000, Wu Xi Kang Ning medical apparatus Co. Ltd., China). Laser ablation was subsequently performed on the stroma under the flap using excimer laser machine (EC-5000CXI, NIDEK, Japan). Animals in the study underwent LASIK on both eyes (Fig. 1). In the first group, only typical LASIK flaps were created without ablation; in the second and third group, the flaps were created and residual stroma bed was 50 or 30% of the whole cornea thickness respectively. Then the new surfaces between the flap and the stroma were rinsed with physiological saline repeatedly. At last, the flap floated back into position, and then two drops of tobramycin solution 0.3% were instilled into the eye. The accuracy of cutting depth was monitored by intraoperative pachymetry.

Rabbits with flaps displaced, visible striae on the first day after surgery, or with eyes infected during the following days were excluded and replacement animals were added.

### Specimen preparation

The central corneal thickness was measured using an ultrasound pachymeter before the animals were killed. All the animals were anaesthetized with ether and humanely killed by air embolism method after 7 or 28 days post-operatively. The cornea tissue was obtained and cut into a rectangular strip with 3.5 mm wide using a commercialized double-edged scalpel. Strip specimens in this study were taken from temporal-nasal direction.

### Biomechanical measurements

**Fig. 2:**
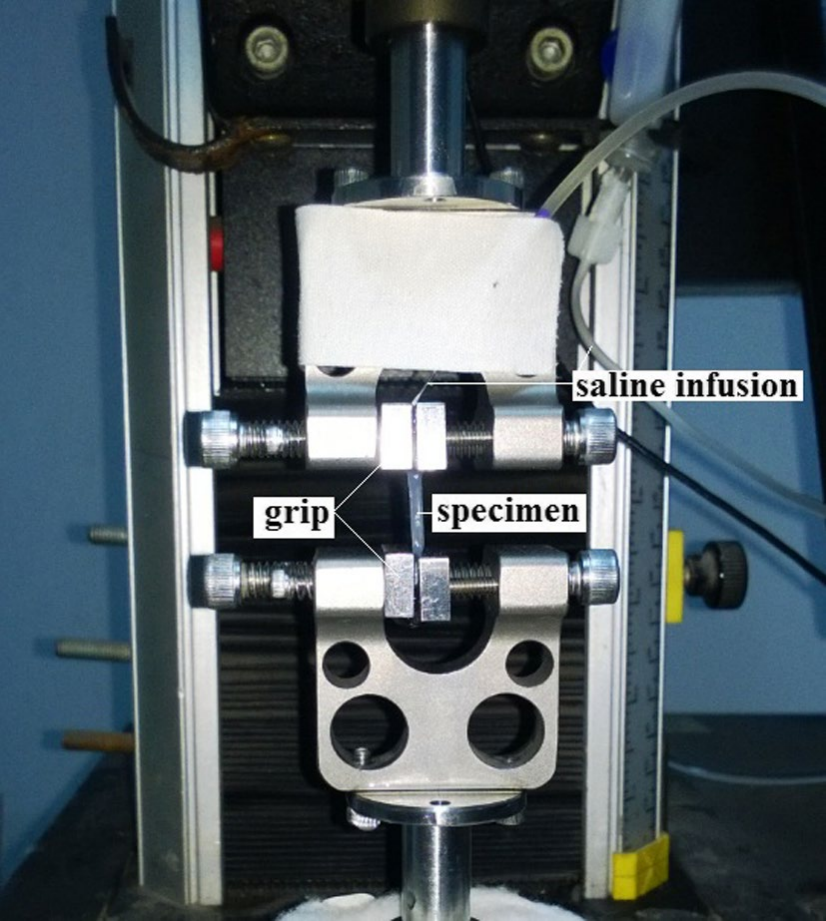
Evaluation of biomechanical properties of corneal strips was carried out on Instron 5544 tester

Uniaxial tensile test was used to determine the biomechanical properties of the cornea by a tester (5544, Instron Co. Ltd., USA) with a 5.0 N full scale load cell (accuracy 2‰). The limbus and the ends of the corneal strip were clamped used for connection to the grips of the tester, leaving the middle corneal tissue to resist the applied tension loads (Fig. 2). Prior to tensile test, for each specimen, the original length *L* (mm) which represented the distance between grips was measured. In order to avoid drying, a saline infusion was fixed on the moving clamp. The load from 0 to 0.05 N were imposed on the specimens at a rate of 2 mm/min. The corresponding data of load (N) and displacement (mm) were recorded by a computer.

**Fig. 3:**
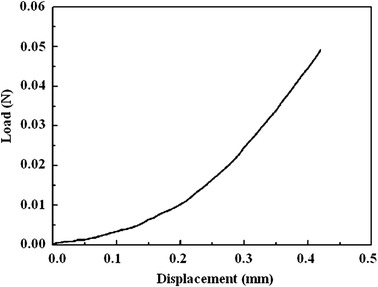
The load–displacement curve of a corneal strip

The physiologic level of intraocular pressure (*p*) in an adult rabbit globe is approximately 15–30 mmHg (2–4 kPa); the radius (*r*) of the adult rabbit cornea is approximately 7.5 mm. The load (*F*) on a corneal with a width (*w*) of 3.5 mm is approximately 0.025–0.05 N, as calculated according to the following formula: *F* = *p* × *r* × *w*/2. Therefore, the tensile load corresponding to the calculated modulus of elasticity was considered to be the physiological modulus [22]. Figure 3 showed the relationship of load–displacement of a normal rabbit cornea. The slope of the load–displacement curve was linear in the range of 0.025–0.05 N.

### The mathematical analysis procedure

A mathematical analysis procedure that seeks to consider and eliminate the effects of the deficiencies of strip testing has been presented by Elsheikh A and Anderson K in determining the material properties of normal corneas [21]. In their model, the thickness difference between the central cornea and the limbus was simplified as a slant. This simplification is feasible for normal cornea when the change of corneal geometrics is not as obvious as the ones after LASIK. The excimer laser for photoablation of the corneal stromal bed alters the anterior corneal thickness. Therefore, the cornea can’t be treated as a uniform thickness material in uniaxial tensile test. In order to remedy the inaccuracy from the thickness variation after LASIK in strip test, a simple mathematical analysis procedure was deduced to mimic the actual geometrics of corneal specimens after LASIK. The cornea is simplified as an isotropic and linear elastic material. The thickness of the normal cornea without LASIK is regarded as homogeneity.

**Fig. 4:**
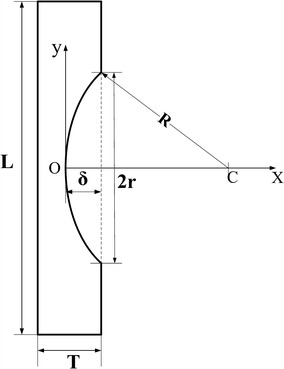
A diagram of the corneal strip after LASIK against an X–Y coordinate system

Figure 4 shows a diagram of the corneal strip after LASIK against an X–Y coordinate system. The origin of coordinate is O. The spherical cap represents the ablated segment with a curvature radius R. The width of the cross-section is constant.

1 $$\left( {x - R} \right)^{2} + y^{2} = R^{2}$$

when *x* = *δ*, *y* = *r*,

2 $$\left\{ \begin{aligned} R &= \frac{{r^{2} + \delta^{2} }}{2\delta } \hfill \\ x &= R - \sqrt {R^{2} - y^{2} } \hfill \\ \end{aligned} \right.$$

where *δ* is the ablated thickness, and *R* is the curvature radius of the ablated area.

For the unabated part, the length of elongation during uniaxial tensile test is calculated by Eq. (3):

3 $$\Delta l_{1} = \frac{\Delta F}{EWT}(L - 2r)$$

where, *L* is the original length of a specimen; *W* and *T* are the width and thickness of the specimen, respectively; 2*r* is the ablated range; *E* is the elastic modulus.For the ablated part, the length of elongation is calculated by Eq. (4):

4 $$\Delta l_{2} = 2\int_{0}^{r} {\frac{\Delta F}{EW(T - \delta + x)}} dy = \frac{2\Delta F}{EW}\int_{0}^{r} {\frac{1}{{T - \delta + R - \sqrt {R^{2} - y^{2} } }}} dy$$

The total length of elongation is expressed as Eq. (5):

5 $$\Delta l = \Delta l_{1} + \Delta l_{2} = \frac{\Delta F}{EW}\left( {\frac{L - 2r}{T} + 2\int_{0}^{r} {\frac{dy}{{{\text{B}} - \sqrt {R^{2} - y^{2} } }}} } \right)$$

where *B* = *T* − *δ* + *r.*

6 $$\begin{aligned} \int_{0}^{r} {\frac{dy}{{B - \sqrt {R^{2} - y^{2} } }}} &= B\left( {\frac{{\arctan \frac{r}{{\sqrt {B^{2} - R^{2} } }}}}{{\sqrt {B^{2} - R^{2} } }} + \frac{{\arctan \frac{Br}{{\sqrt {B^{2} - R^{2} } \sqrt {R^{2} - r^{2} } }}}}{{\sqrt {B^{2} - R^{2} } }}} \right)\\ &\quad - \arctan \frac{r}{{\sqrt {B^{2} - R^{2} } }}\end{aligned}$$

7 $$\begin{aligned} E &= \frac{\Delta F}{\Delta lW}\left[ \frac{L - 2r}{T} + 2B\left( \frac{ {\text{arctan}} \frac{r}{ \sqrt{B^{2} - R^{2}} } }{ \sqrt{B^{2} - R^{2}} } + \frac{ {\text{arctan}} \frac{Br}{ \sqrt{B^{2} - R^{2}} \sqrt{R^{2} - r^{2}} }}{ \sqrt{B^{2} - R^{2}} } \right)\right.\\ &\quad \left. - {\text{arctan}} \frac{r}{ \sqrt{B^{2} - R^{2} } } \right] \end{aligned}$$

Equation (7) is used to determining the elastic modulus of corneal strips after LASIK. In this study, the slope of the load–displacement curve (Δ*F*/Δ*F*) was nearly linear in the range of 0.025–0.05 N (approximately as the physiologic level of intraocular pressure 15–30 mmHg), thus the corneal mechanical behavior presented here was regarded as linear elasticity. All parameters were presented in Table 1.

**Table 1:** Central corneal thickness (CCT) and ablation depth (*δ*) of the cornea

	CCT (μm)				*δ* (μm)
	Before LASIK	0 day after LASIK	7 days after LASIK	28 days after LASIK	
Control	402 ± 29	398 ± 33	394 ± 27	401 ± 24	0
Group 1	379 ± 36	382 ± 32	386 ± 23	390 ± 34	
Group 2	394 ± 19	287 ± 29	279 ± 25	286 ± 23	107 ± 18
Group 3	408 ± 38	212 ± 23	216 ± 28	208 ± 21	196 ± 27

### Statistical analysis

All data were expressed as mean ± standard deviation. Statistical analysis was performed using one-way ANOVA analysis followed by Post hoc Tukey testing. The level of significance was considered when *P* < 0.05.

## Results

**Fig. 5:**
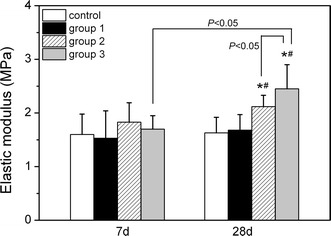
Effects of variation in ablation depth on the corneal elastic modulus after LASIK. *P < 0.05, compared to normal control after 28 days post-operatively; ^#^P < 0.05, compared to group 1 after 28 days post-operatively. n = 10 in each group

The elastic modulus was about 1.60 MPa in normal controls when the loading force ranged from 0.025 N to 0.05 N. After 7 days, there were no significant differences among four groups examined (Control, 1.60 ± 0.38 MPa; group 1, 1.53 ± 0.51 MPa; group 2, 1.83 ± 0.36 MPa; group 3, 1.70 ± 0.25 MPa). After 28 days, no obvious differences were found between the normal control (1.60 ± 0.38 MPa) and group 1 (1.68 ± 0.29 MPa) (*P* > 0.05). Compared to the normal control, there was a significant increase in the elastic modulus of corneal strips in group 2 (2.12 ± 0.21 MPa) by 30% or group 3 (2.45 ± 0.45 MPa) by 51% (*P* < 0.05). Compared to the group 1, there was a significant increase in the elastic modulus of corneal strips in group 2 by 26% or group 3 by 46% (*P* < 0.05). The elastic modulus of corneal strips in group 3 increased by 16% compared to group 2 (*P* < 0.05), this indicated that the corneal elastic modulus increased with the ablation depth after 28 days post-operatively (Fig. 5).

This data also showed that corneal strips in group 3 after 28 days had a higher elastic modulus than those of 7 days post-operatively (*P* < 0.05); while there was no significant differences in other groups (*P* > 0.05).

## Discussion

Animal models allow research to be conducted under controlled parameters to remedy limitations in humans. In this study, we investigated the effects of ablation depth and repair time on the corneal biomechanical properties after LASIK. Our results found that there was no significant difference in corneal elastic modulus after 7 days post-operatively among the four groups. 28 days post-operatively, the corneal elastic modulus increased in ablation depth-dependent manner, and the cornea with 30% residual stroma bed increased elastic modulus by 41% compared to that of 7 days. Thus, alteration in corneal biomechanics after LASIK may correlate with the ablation depth and repair time. The biomechanical response of the cornea after LASIK is dominated by the stroma. Flap creation did not significantly alter the elastic modulus of the cornea. In addition, the mathematical procedure presented here is more reasonable in determining the corneal material properties after LASIK, and improves the accuracy of corneal strip tests.

The cornea is composed of five layers, and considered as a complex anisotropic composite with non-linear elastic and viscoelastic properties in term of material science. However, most biological soft tissues approximate linear elastic behavior when a small range of stresses is considered. In this study, the slope of the load–displacement curve was nearly linear in the range of 0.025–0.05 N (approximately as the physiologic level of intraocular pressure 15–30 mmHg), thus the corneal mechanical behavior presented here was regarded as linear elasticity. The elastic modulus provides an intrinsic indicator of material stiffness. A high modulus indicates a stiff or low-compliance material. In this study, the creation of a thin 90–110 μm flap did not additionally compromise the elastic modulus of the cornea. There was also no significant change in CRF or CH after flap creation [23], and minimal biomechanical loading is distributed through the flap [14]. However, there was significant decrease in these parameters following laser ablation, when compared to values obtained pre-operatively. All these findings indicate that, the mechanical response of the cornea after LASIK may be predominantly due to laser ablation.

The strip test was also used by Fang et al. [20] to determine the corneal elastic modulus of the porcine eyes after LASIK. Their results showed that the corneal elastic modulus decreased with ablation depth. The anterior 40% of the central corneal stroma is the strongest region of the cornea, whereas the posterior 60% of the stroma is at least 50% weaker [24]. This biomechanical effect correlated with deeper ablation may be due to more central collagen and matrix material removed [6, 25]. The differences between Fang’ study and ours may stem from that they measured the corneal elastic modulus immediately after LASIK in vitro, the cornea didn’t undergo wound healing process.

As is known, when the cornea is injured, a series of complex and coordinated cellular processes are activated to achieve the corneal tissue wound healing. The process of remodeling and repair begins at 48 h, peaks between several days and 1 month after injury, and is sustained for 3 months or even several years [26]. In this study, the elastic modulus increasing with ablation depth after 28 days post-operatively may be explained by Davis’s law, which is used to describe how soft tissue models along imposed demands. It is a physiological principle stating that soft tissue heal according to the manner in which they are mechanically stressed [27]. It is the corollary to Wolff’s law, which applies to osseous tissue. It is feasible that the increased postoperative load born by the residual stroma signals a proliferative keratocyte response aimed at increasing structural resistance to this stress [1]. Therefore, we speculate that over a period of wound healing, the residual stroma with deep ablation may become stronger to combat the increasing mechanical stress without failing within short time after surgery.

Data from Liu [28] showed that the nonlinear mechanical behavior of the cornea was closely correlated with the crimping morphology of collagen fibrils. The straightened fibrils, acting as reinforcing frames, enhance the corneal mechanical properties until they break. After LASIK, the central corneal tissue becomes relative flatten and thin, and the cornea is subjected to greater tension stress than pre-operation in the same IOP. The more the volume is ablated, the greater is the tensile stress in the remained cornea. The collagen fibrils of the cornea may adjust with respect to this condition. Elastic modulus increasing with ablation depth after 28 days post-operatively may also be explained that, the fibrils from the remained cornea may become straighter under the greater tensile stress, thus corneal mechanical properties may also be enhanced. However, low residual stromal bed thickness from excessive ablation is a risky factor for post-LASIK keratectasia, which may show itself immediately or many months after LASIK but generally occurs within 2 years [29]. The long-term effect of excessive ablation on the corneal material properties is still unclear and should be further investigated.

Previous studies have suggested CH and the CRF decreased significantly post-operatively. CRF is dominated by the elastic properties of the cornea and appears to be an indicator of the overall ‘resistance’ of the cornea, that is, it reflects the overall biomechanical effects of the specimen (considering the dimension of the cornea), not material property itself. For example, whereas corneas from patients with keratoconus are generally thinner than average and biomechanically floppy, corneas from patients with Fuchs dystrophy are thicker than normal while also being biomechanically similar to keratoconus [5, 30]. Thus, although the elastic modulus becomes larger with ablation depth, the thickness decreases more remarkably than the elastic modulus, the cornea will be more readily deformable with excessive ablation.

Our study is limited by its small sample of eyes studied and is in the absence of long-term data. Another limitation of the study is that the determination of the corneal elastic modulus is carried out in vitro, and the corneal tissue is simplified as an isotropic and homogeneous material. Differences exist in the collagen fibers arrangement among the anterior, middle and posterior of corneal stroma [31, 32]. The structural difference inside the tissue may influence the mechanical properties of the cornea. In addition, Differences in corneal structure [33] and wound healing response also exist between mammalian. As a consequence of mentioned reasons, one should be cautious in extrapolating the data from rabbits to human cases.

## Conclusions

The biomechanical responses of the cornea after LASIK are linked in repair time and ablation depth. Complex corneal biomechanical processes influence the integrity of the normal and postoperative cornea, and developing an understanding of these processes may facilitate recognition of risk factors for ectasia after LASIK.

## References

[1] Dupps WJ, Wilson SE (2006). Biomechanics and wound healing in the cornea. Exp Eye Res.

[2] Piñero DP, Alcón N (2015). Corneal biomechanics: a review. Clin Exp Optom.

[3] Tatar MG, Aylin Kantarci F, Yildirim A, Uslu H, Colak HN, Goker H, Gurler B (2014). Risk factors in post-LASIK corneal ectasia. J Ophthalmol.

[4] Yang E, Roberts CJ, Mehta JS (2016). A review of corneal biomechanics after LASIK and SMILE and the current methods of corneal biomechanical analysis. Clin Exp Ophthalmol.

[5] Luce DA (2005). Determining in vivo biomechanical properties of the cornea with an ocular response analyzer. J Cataract Refract Surg.

[6] Ortiz D, Piñero D, Shabayek MH, Arnalich-Montiel F, Alinalich-Montiel F, Alió JL (2007). Corneal biomechanical properties in normal, post-laser in situ keratomileusis, and keratoconic eyes. J Cataract Refract Surg.

[7] Pedersen IB, Bak-Nielsen S, Vestergaard AH, Ivarsen A, Hjortdal J (2014). Corneal biomechanical properties after LASIK, ReLEx flex, and ReLEx smile by Scheimpflug-based dynamic tonometry. Graefes Arch Clin Exp Ophthalmol.

[8] Wang D, Liu M, Chen Y, Zhang X, Xu Y, Wang J, To CH, Liu Q (2014). Differences in the corneal biomechanical changes after SMILE and LASIK. J Refract Surg.

[9] Chen S, Chen D, Wang J, Lu F, Wang Q, Qu J (2010). Changes in ocular response analyzer parameters after LASIK. J Refract Surg.

[10] He R, Zhou YX, Niu JM (2012). A study of the corneal biomechanical characteristics in the preoperative and postoperative periods of laser in situ keratomileusis. Chin J Optom Ophthalmol Vis Sci.

[11] Qazi MA, Sanderson JP, Mahmoud AM, Yoon EY, Roberts CJ, Pepose JS (2009). Postoperative changes in intraocular pressure and corneal biomechanical metrics: laser in situ keratomileusis versus laser-assisted subepithelial keratectomy. J Cataract Refract Surg.

[12] Zu PP, Wang Y, Wu D, Wei SS (2013). Short-term influence of different refractive surgery on corneal biomechanical properties. Chin J Pract Ophthalmaol.

[13] Förster W, Stupp T, Kasprzak H (2003). Hoolographic interferometry of excimer-laser-ablated bovine eyes: first results. Ophthalmologica.

[14] Jaycock PD, Lobo L, Ibrahim J, Tyrer J, Marshall J (2005). Interferometric technique to measure biomechanical changes in the cornea induced by refractive surgery. J Cataract Refract Surg.

[15] Knox Cartwright NE, Tyrer JR, Jaycock PD, Marshall J (2012). Effects of variation in depth and side cut angulations in LASIK and thin-flap LASIK using a femtosecond laser: a biomechanical study. J Refract Surg.

[16] Andreassen TT, Simonsen AH, Oxlund H (1980). Biomechanical properties of keratoconus and normal corneas. Exp Eye Res.

[17] Nash IS, Greene PR, Foster CS (1982). Comparison of mechanical properties of keratoconus and normal corneas. Exp Eye Res.

[18] Seiler T, Matallana M, Sendler S, Bende T (1982). Does Bowman’s layer determine the biomechanical properties of the cornea?. Refract Corneal Surg.

[19] Borja D, Manns F, Lamar P, Rosen A, Fernandez V, Parel JM (2004). Preparation and hydration control of corneal tissue strips for experimental use. Cornea.

[20] Fang XJ, Xu YC (2006). Corneal stresss-strain relation and structural equation of procine eye after LASIK. Int J Ophthalmaol.

[21] Elsheikh A, Anderson K (2005). Comparative study of corneal strip extensometry and inflation tests. J R Soc Interface.

[22] Chen WY, Wang XJ, Wang CY, Li T, Li XN, Zhang QY (2008). An experimental study on collagen content and biomechanical properties of sclera after posterior sclera reinforcement. Clin Biomech.

[23] Uzbek AK, Kamburoğlu G, Mahmoud AM, Roberts CJ (2011). Change in biomechanical parameters after flap creation using the Intralase femtosecond laser and subsequent excimer laser ablation. Curr Eye Res.

[24] Randleman JB, Dawson DG, Grossniklaus HE, McCarey BE, Edelhauser HF (2008). Depth-dependent cohesive tensile strength in human donor corneas: implications for refractive surgery. J Refract Surg.

[25] Garcia-Porta N, Fernandes P, Queiros A, Salgado-Borges J, Parafita-Mato M, González-Méijome JM (2014). Corneal biomechanical properties in different ocular conditions and new measurement techniques. ISRN Ophthalmol..

[26] Netto MV, Mohan RR, Ambrósio R, Hutcheon AE, Zieske JD, Wilson SE (2005). Wound healing in the cornea: a review of refractive surgery complications and new prospects for therapy. Cornea.

[27] Ellenbecker TS, Carlo MD, Derosa C. Effective functional progressions in sport rehabilitation. Hum Kinet. 2009.

[28] Liu X, Wang L, Ji J, Yao W, Wei W, Fan J, Joshi S, Li D, Fan YA (2014). Mechanical model of the cornea considering the crimping morphology of collagen fibrils. Invest Ophthalmol Vis Sci.

[29] Comaish IF, Lawless MA (2002). Progressive post-LASIK keratectasia biomechanical instability or chronic disease process. J Cataract Refract Surg.

[30] Pepose JS, Feigenbaum SK, Qazi MA, Sanderson JP, Roberts CJ (2007). Changes in corneal biomechanics and intraocular pressure following LASIK using static, dynamic, and noncontact tonometry. Am J Ophthalmol.

[31] Komai Y, Ushiki T (1991). The three-dimensional organization of collagen fibrils in the human cornea and sclera. Invest Ophthalmol Vis Sci.

[32] Meek KM, Boote C (2009). The use of X-ray scattering techniques to quantify the orientation and distribution of collagen in the corneal stroma. Prog Retin Eye Res.

[33] Hayes S, Boote C, Lewis J, Sheppard J, Abahussin M, Quantock AJ, Purslow C, Votruba M, Meek KM (2007). Comparative study of fibrillar collagen arrangement in the corneas of primates and other mammals. Anat Rec.

